# Metabolic syndrome biomarkers relate to rate of cognitive decline in MCI and dementia stages of Alzheimer’s disease

**DOI:** 10.1186/s13195-023-01203-y

**Published:** 2023-03-16

**Authors:** Jagan A. Pillai, James Bena, Lynn Bekris, Nandan Kodur, Takhar Kasumov, James B. Leverenz, Sangeeta R. Kashyap, on behalf of the Alzheimer’s Disease Neuroimaging Initiative

**Affiliations:** 1 Lou Ruvo Center for Brain Health, Cleveland Clinic Lerner College of Medicine, Case Western Reserve University, 9500 Euclid Ave/U10, Cleveland, OH 44195 USA; 2 Neurological Institute, Cleveland Clinic Lerner College of Medicine, Cleveland, OH 44195 USA; 3 Department of Neurology, Cleveland Clinic Lerner College of Medicine, Cleveland, OH 44195 USA; 4 Cleveland Clinic Lerner College of Medicine, Cleveland, OH 44195 USA; 5 Quantitative Health Sciences, Cleveland Clinic Lerner College of Medicine, Cleveland, OH 44195 USA; 6 Lerner Research Institute, Cleveland Clinic Lerner College of Medicine, Cleveland, OH 44195 USA; 7 Department of Pharmaceutical Sciences, Northeast Ohio Medical University, Rootstown, OH 44272 USA; 8 Division of Endocrinology, Diabetes and Metabolism, Weill Cornell Medicine New York Presbyterian, New York, NY 10021 USA

**Keywords:** Alzheimer’s disease, Metabolic syndrome, ApoA1, Triglyceride/high-density lipoprotein ratio, Cognitive decline, MCI, Dementia, Blood–brain barrier, Neuroinflammation, CSF, Apolipoprotein

## Abstract

**Background:**

The relationship between biomarkers of metabolic syndrome and insulin resistance, plasma triglyceride/HDL cholesterol (TG/HDL-C) ratio, on the rate of cognitive decline in mild cognitive impairment (MCI) and dementia stages of Alzheimer’s disease (AD) is unknown. The role of peripheral and cerebrospinal fluid (CSF) levels of Apolipoprotein A1 (ApoA1), a key functional component of HDL, on cognitive decline also remains unclear among them. Here we evaluate baseline plasma TG/HDL-C ratio and CSF and plasma ApoA1 levels and their relation with cognitive decline in the MCI and Dementia stages of AD.

**Patients and methods:**

A retrospective longitudinal study (156 participants; 106 MCI, 50 AD dementia) from the Alzheimer’s Disease Neuroimaging Initiative, with an average of 4.0 (SD 2.8) years follow-up. Baseline plasma TG/HDL-C, plasma, and CSF ApoA1 and their relationship to inflammation and blood–brain barrier (BBB) biomarkers and longitudinal cognitive outcomes were evaluated. Multivariable linear mixed effect models were used to assess the effect of baseline analytes with longitudinal changes in Mini-Mental State Exam (MMSE), Clinical Dementia Rating–Sum of Boxes (CDR-SB), and Logical Memory delayed recall (LM) score after controlling for well-known covariates.

**Results:**

A total of 156 participants included 98 women, 63%; mean age was 74.9 (SD 7.3) years. At baseline, MCI and dementia groups did not differ significantly in TG/HDL-C (Wilcoxon *W* statistic = 0.39, *p* = 0.39) and CSF ApoA1 levels (*W* = 3642, *p* = 0.29), but the dementia group had higher plasma ApoA1 than the MCI group (*W* = 4615, *p* = 0.01). Higher TG/HDL-C ratio was associated with faster decline in CDR-SB among MCI and dementia groups. Higher plasma ApoA1 was associated with faster decline in MMSE and LM among MCI, while in contrast higher CSF ApoA1 levels related to slower cognitive decline in MMSE among MCI. CSF and plasma ApoA1 also show opposite directional correlations with biomarkers of BBB integrity. CSF but not plasma levels of ApoA1 positively correlated to inflammation analytes in the AGE-RAGE signaling pathway in diabetic complications (KEGG ID:KO04933).

**Conclusions:**

Biomarkers of metabolic syndrome relate to rate of cognitive decline among MCI and dementia individuals. Elevated plasma TG/HDL-C ratio and plasma ApoA1 are associated with worse cognitive outcomes in MCI and dementia participants. CSF ApoA1 and plasma ApoA1 likely have different roles in AD progression in MCI stage.

**Supplementary Information:**

The online version contains supplementary material available at 10.1186/s13195-023-01203-y.

## Introduction

Metabolic syndrome refers to the co-occurrence of cardiovascular risk factors, including insulin resistance, obesity, dyslipidemia, and hypertension, and identifies patients who are at high risk of developing atherosclerotic cardiovascular disease and type 2 diabetes [1]. Meta-analyses have reported that those with diabetes diagnosis have a 25–91% elevated risk of developing dementia in their lifetimes [2], while metabolic syndrome increased the risk of progression from mild cognitive impairment (MCI) to dementia [3]. Cardiovascular risk factors and diabetes are also well-known modifiable factors for dementia risk worldwide [4]. However, the relationship between biomarkers of metabolic syndrome on the rate of cognitive decline among MCI and dementia patients is still poorly characterized.

Plasma triglyceride-to-HDL cholesterol (TG/HDL-C) is often routinely measured in general clinical practice and is often elevated in individuals with metabolic syndrome [5, 6]. High TG/HDL-C is the single most powerful predictor of extensive coronary heart disease among other lipid variables [7]. High TG/HDL-C has been reported to be closely related to systemic insulin resistance and subclinical inflammation in adults [8, 9]. However, there is conflicting information concerning the association of TGs on Alzheimer’s disease (AD) and its related outcomes [10–13], as the changes observed could be related to disease stage. Imaging studies have shown that higher insulin resistance and subsequent hyperinsulinemia predict hypermetabolism in the early mild cognitive impairment (MCI) stage clinical progressors and hypometabolism in medial temporal regions in the later AD dementia stage [14]. Clarifying the role of TG/HDL-C changes in both MCI and AD dementia stages is important for targeted lifestyle and metabolic syndrome modifying therapies to improve cognitive outcomes.

Elevated plasma high-density lipoproteins (HDL) levels have been reported to associate with sharper mental abilities in the elderly [9, 10], while low HDL levels are a risk factor for future memory decline in middle-aged adults [11]. Apolipoprotein A1 (ApoA1) is the key functional component of HDL that participates in cholesterol efflux. *APOA1* gene polymorphism has also been related to cognitive impairment [15]. ApoA1 is anti-atherogenic and reduces vascular inflammation and thus has protective effects on cardiovascular and stroke risk in addition to amyloid changes related to AD [16]. Plasma ApoA1 is a stronger prognostic marker than HDL cholesterol for cardiovascular disease and ischemic stroke in some studies [17, 18]. ApoA1 has robust animal and clinical data supporting its role in AD pathophysiology [19, 20]. Lower plasma ApoA1 and HDL levels correlate with higher amyloid burden, increased risk for AD [21, 22], and increased cognitive score severity in AD [22]. However, among normal cognition elderly subjects, the association between plasma ApoA1 and the risk of dementia is inconclusive in results from prospective and cohort studies [23–26].

Cells in the brain do not express ApoA1, and plasma ApoA1 enters the central nervous system (CNS) from the periphery to become a component of CNS lipoproteins [27]. Results from AD mouse models suggest that ApoA1 may be regulated by distinct mechanisms on either side of the blood–brain barrier (BBB) and that ApoA1 may serve to integrate peripheral and CNS lipid metabolism [28]. The relative contributions of peripheral and CNS pools of ApoA1 to neuropathological and behavioral outcomes and their association with BBB integrity is controversial [16]. However, clarity on this association is important for potential targeted therapeutic interventions against ApoA1 mechanisms in clinical patients with AD.

This study has two primary aims: (1) to address our lack of understanding of the relationship between a commonly used clinical biomarker of metabolic syndrome, plasma TG/HDL-C ratio, and cognitive outcomes in the MCI or dementia stages of AD, and (2) given that increased BBB permeability and related biomarker changes have been reported in MCI and dementia stages of AD [29], to clarify the relationship between plasma or cerebrospinal fluid (CSF) ApoA1 levels and clinical outcomes as well as evaluate their levels in relation to BBB integrity biomarkers. Our main hypotheses were as follows: (a) that plasma TG/HDL-C ratio is a clinical biomarker that associates with measures of longitudinal cognitive decline in MCI and dementia and (b) both plasma and CSF levels of ApoA1 equally associate with measures of longitudinal cognitive decline in MCI and dementia. We addressed these hypotheses among MCI and dementia participants in the Alzheimer’s Disease Neuroimaging Initiative (ADNI) cohort.

## Methods

### Patients

The ADNI is a longitudinal multicenter study designed to develop clinical, imaging, genetic, and biochemical biomarkers for the early detection and tracking of AD. ADNI was launched by the National Institute of Aging with additional support from private pharmaceutical companies and nonprofit organizations. The eligibility criteria for the first phase of the ADNI study are described in the ADNI1 protocol (http://adni.loni.usc.edu/methods/documents/). Briefly, eligible participants (55 to 90 years old) had an informant that provided an independent evaluation of functioning and was fluent in either English or Spanish. Participants had completed at least 6 years of education (or had a work history sufficient to exclude intellectual disability). For clinical staging, the categories of MCI (MCI group) and AD dementia (dementia group) were used as noted in the ADNIMERGE dataset (downloaded on 10/05/2020). AD biomarker concentration data in CSF, including CSF Aβ1–42, t-tau, and p-tau181, were generated using the Elecsys method [30] provided in ADNIMERGE. Previously published CSF Aβ1–42 and p-tau181 cut points in the ADNI sample, Aβ1–42 < 976.6 pg/mL, p-tau181 > 21.8 pg/mL, were used to define amyloid-positive and tau-positive frequency in the MCI and dementia groups [31].

### CSF and plasma ApoA1 along with BBB biomarkers

In ADNI, levels of ApoA1 and BBB biomarkers (Vascular cell adhesion protein 1, VCAM1; Intercellular Adhesion Molecule 1, ICAM1; and Matrix metallopeptidase 9, MMP9) were assessed in a fasting state with the RBM Discovery MAP® v.1.0 panel, on a Luminex platform (Myriad Genetics; Salt Lake City, UT). The CSF and plasma multiplex data used in this analysis were cleaned and quality controlled based on the methodology described in the statistical analysis of the Biomarkers Consortium Data Primer. The least detectable level for ApoA1, VCAM1, ICAM1, and MMP9 was 0.006875 μg/ml, 0.0148 ng/ml, 0.84 ng/ml, and 4.08 ng/ml, respectively.

### Inflammatory biomarkers

We explored the relationship between ApoA1 and a panel of CSF and plasma inflammatory biomarker data in ADNI participants who had curated CSF and plasma multiplex data (see Supplementary material), the details of which have been published previously [32].

### Plasma TG/HDL-C

Total TG and HDL-C data was downloaded from the ADNI_LIPIDOMICSRADER file on 12/15/2021. Total TG and HDL-C were determined using COBAS C311 analyzer assays (Roche Diagnostics, Florham Park, NJ), which utilize enzymatic colorimetric methods. These analysis methods are further described in ADNI reference documents with plasma tests performed after overnight fasting (http://adni.loni.usc.edu/methods/documents/).

### Cognitive and functional measures

The Mini-Mental State Exam (MMSE) [33], Clinical Dementia Rating–Sum of Boxes (CDR-SB) [34], and Logical Memory delayed recall score (LM) (Wechsler Memory Scale, Logical Memory subtest) [35] were used to characterize the degree of baseline cognitive and functional deficits and to assess cognitive and functional change from baseline at each year of longitudinal follow-up.

### Body mass index (BMI), lipid-modifying medications, and diabetes status

Participant BMI ranged from 17.2 to 38.8, mean 25.5 (3.7). A history of use of lipid profile modifying medications (see Supplementary material) was noted among 40 of the 156 participants at the time of plasma and CSF measurements. The presence of type II diabetes among participants was ascertained from their medical history and included 6 of the 156 participants.

### Statistical analysis

Normality was assessed for continuous variables by Q-Q plots following which a *t*-test was applied to compare normally distributed continuous variables. Wilcoxon rank-sum test with *W* statistic was applied to compare non-normally distributed continuous variables. Chi-squared test was conducted for categorical variables. Multivariable linear mixed effect models were used to assess the effect of baseline plasma TG/HDL-C ratio, plasma ApoA1, and CSF ApoA1 when taken together with changes in CDR-SB and MMSE (dependent variable) after controlling for well-known covariates of age, sex, education years, BMI, CSF Abeta-42, CSF p-tau, use of lipid-lowering medications, and *APOEε4* status. Multiple imputation using fully conditional specification was performed to account for missing data in covariates used in the multivariable models (missing data in BMI, CSF p-tau variables) and 10 imputation data sets were created. Models were run separately for each imputation data set and then pooled to provide sensitivity analysis. Additional exploratory analyses evaluated whether associations between baseline markers and changes in cognitive outcomes within strata were defined by *APOEε4* status. Univariate Pearson correlations between the inflammatory markers and TG/HDL and ApoA1 with a false discovery rate (FDR) correction of 0.05 were also completed. The sample size within each stratum prevented evaluation of interactions between *APOEε4* status and the other markers and for the role of diabetes in these results.

All tests were two-tailed and with a significance level of  < 0.05. R version 3.5.1 (The R Foundation for Statistical Computing, Vienna, Austria), SAS software version 9.4 (SAS, Cary, NC), and IBM SPSS Statistics for Windows, Version 22.0. (IBM, Armonk, NY) were used for analyses.

### Functional pathway analysis on inflammatory analytes of interest

The inflammatory analytes of significance identified in the univariate correlational analysis in relation to TG/HDL, plasma, and CSF ApoA1 were entered into STRING: functional protein association networks for pathway enrichment analysis [36]. The top pathway for each analysis with the largest analyte count and the lowest *P* value following false discovery rate (FDR) correction is reported.

## Results

A total of 156 participants were included in the analysis [MCI group (*n* = 106) and Dementia group (*n* = 50); 98 women, 63%; mean (SD) age was 74.9 (7.3) years]. 64 of 106 in the MCI group (60.3%) and 37 of 50 in the dementia group (74%) were CSF amyloid and tau positive supporting underlying AD pathology. Table 1 provides demographics, baseline cognitive data, and biomarker data of participants from the ADNI cohort. At baseline, the MCI and dementia groups did not differ significantly for TG/HDL (*W* = 0.39, *p* = 0.39) and CSF ApoA1 levels (*W* = 3642, *p* = 0.29), but the dementia group had higher plasma ApoA1 than the MCI group (*W* = 4615, *p* = 0.01). Among MCI, plasma/CSF ApoA1 ratio was 744.96 (SD 432.92) and dementia 957.472 (SD 606.822). The TG/HDL ratio correlated moderately with BMI (*r* = 0.33; *p* = 0.002 for MCI; *r* = 0.35; *p* = 0.070 for Dementia). CSF ApoA1 was positively correlated to CSF Abeta 42 levels in MCI (*r* = 0.36, *p* < 0.001) and had borderline significance for dementia (*r* = 0.32, *p* = 0.069) but not p-tau181 or total-tau. TG/HDL and plasma ApoA1 were not significantly correlated to CSF AD biomarkers (Supplementary tables 1 and 2).

**Table 1:** Participant demographics

	**Overall (*N* = 156)**		**MCI group (*N* = 106)**		**Dementia group (*N* = 50)**		**Test**	
**Factor**	***N***	**Statistics**	***N***	**Statistics**	***N***	**Statistics**	**Statistic**	***p*-value**
Age (years)	156	74.9 ± 7.3	106	75.0 ± 6.9	50	74.5 ± 8.0	0.41	0.68^a^
Gender	156		106		50		3.69	0.055^c^
Male		58 (37.2)		34 (32.1)		24 (48.0)		
Female		98 (62.8)		72 (67.9)		26 (52.0)		
BMI	122	25.5 ± 3.7	84	25.6 ± 3.7	38	25.4 ± 3.8	0.22	0.82^a^
Education (years)	156	16.0 [14.0, 18.0]	106	16.0 [14.0, 18.0]	50	15.5 [12.0, 18.0]	3291	***0.016***^***b***^
*APOE ε4* carrier	156	94 (60.3)	106	58 (54.7)	50	36 (72.0)	4.24	***0.040***^***c***^
MMSE	156	25.8 ± 2.4	106	26.9 ± 1.7	50	23.5 ± 1.9	10.8	**< *0.001***^***a***^
CDR-SB	156	2.4 ± 1.7	106	1.6 ± 0.89	50	4.3 ± 1.6	-11.2	**< *0.001***^***a***^
Logical memory delayed recall	156	2.0 [0.00, 4.0]	106	3.0 [1.00, 6.0]	50	0.00 [0.00, 2.0]	2595	**< *0.001***^***b***^
CSF A-Beta42 (log2)*	156	650.5 [497.5, 862.6]	106	665.3 [565.2, 957.8]	50	545.7 [430.7, 730.9]	3162	***0.004***^***b***^
CSF P-tau(log2)*	155	31.3 [22.5, 40.4]	106	29.5 [21.8, 39.1]	49	33.3 [26.0, 44.3]	4250	0.10^b^
Type 2 diabetes medical history	156	6 (3.84)	106	5 (4.71)	50	1 (2)	0.68	0.37^c^
Any lipid-modifying medication use	156	40 (25.6)	106	26 (24.5)	50	14 (28.0)	0.21	0.64^c^
Plasma TG/HDL	156	2.1 [1.3, 3.2]	106	2.0 [1.2, 3.3]	50	2.3 [1.4, 3.2]	4152	0.39^b^
Plasma ApoA1 µg/ml	156	0.56 [0.46, 0.73]	106	0.55 [0.41, 0.71]	50	0.59 [0.52, 0.86]	4615	***0.010***^***b***^
CSF ApoA1 µg/ml	156	0.0008 [0.0006, 0.0012]	106	0.0008 [0.0006, 0.0012]	50	0.0007 [0.0006, 0.0011]	3642	0.29^b^
Follow-up (years)	156	4.0 ± 2.8	106	4.9 ± 3.0	50	2.0 ± 0.5	9.8	**< *0.001***^***a***^

Statistics presented as mean ± SD, median [P25, P75], *N* (column %)

*p*-values: ^a^*t*-test with *T* statistic, ^b^Wilcoxon rank-sum test with *W* statistic, ^c^Pearson’s chi-square test with chi-square statistic

Bold *p* values: *p* < 0.05

^*^60.3% of the MCI group and 74% of the dementia group met CSF amyloid and tau thresholds supporting underlying AD pathology

### Hypothesis 1: Plasma TG/HDL-C ratio and longitudinal cognitive decline

**Fig. 1:**
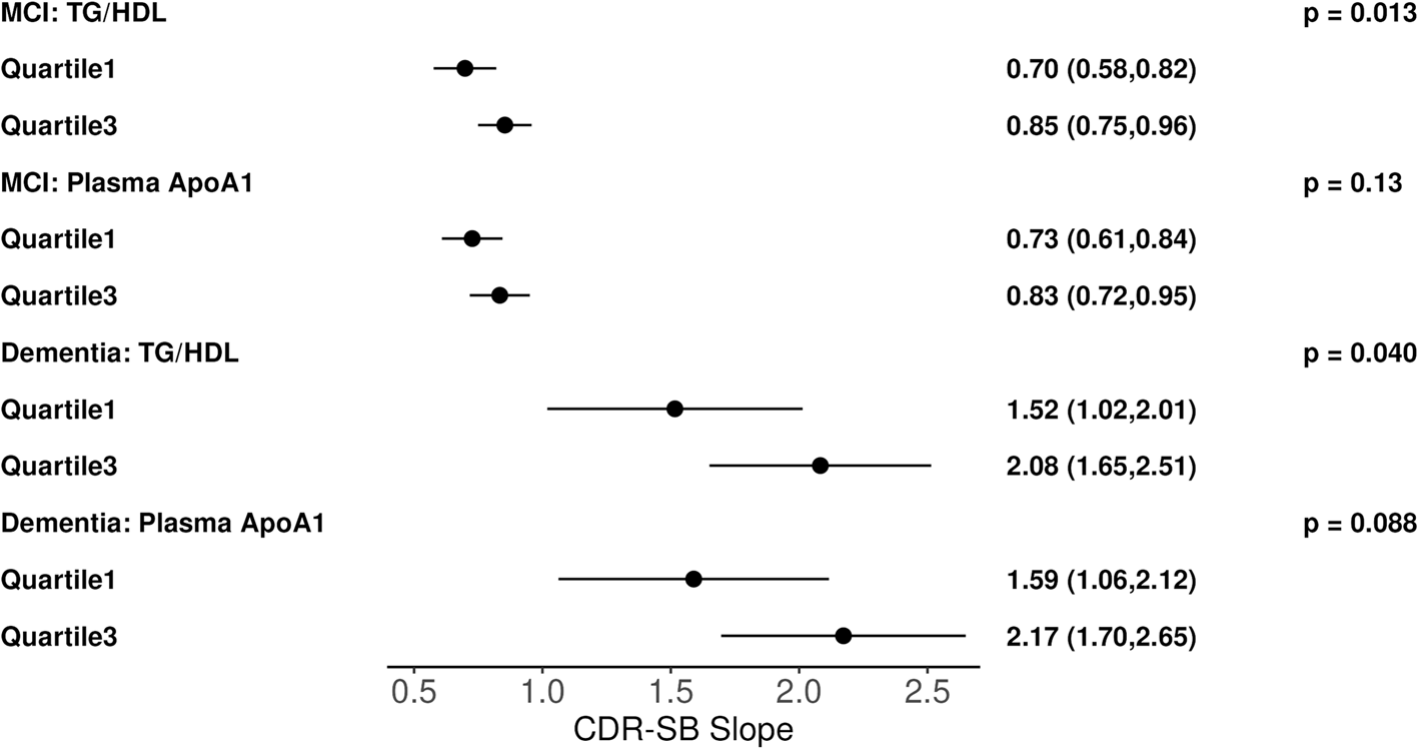
Illustrative plots representing multivariable linear mixed effect model slope and 95% confidence interval results for analytes (TG/HDL, plasma APOA1) versus longitudinal CDR-SB score at the first-quartile and third quartiles in the MCI group (*n* = 106) or dementia group (*n* = 50)

**Fig. 2:**
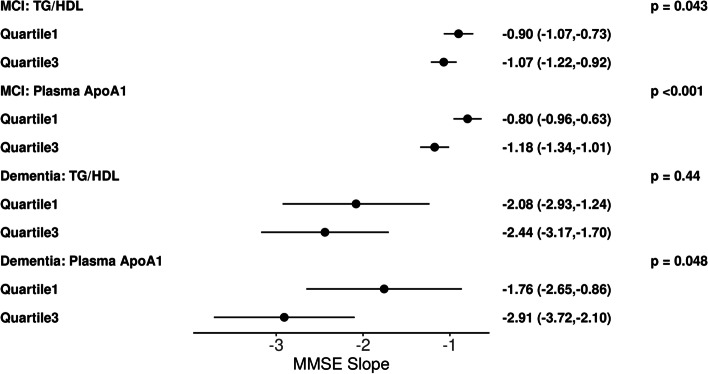
Illustrative plots representing multivariable linear mixed effect model slope and 95% confidence interval results for analytes (TG/HDL, plasma APOA1) versus longitudinal MMSE score at the first quartile and third quartiles in the MCI group (*n* = 106) or dementia group (*n* = 50)

Among subjects in the MCI group, baseline plasma TG/HDL-C ratio was associated significantly with longitudinal change in MMSE (β − 1.13 [95% CI, − 0.25 to − 0.004], *p* = 0.043) and CDR-SB (β 0.12 [95% CI 0.02 to 0.21], *p* = 0.013), where higher TG/HDL-C had a faster rate of decline. Subjects in the dementia group also showed plasma TG/HDL-C ratio to be associated significantly with longitudinal change in CDR-SB (β 0.47 [95% CI 0.02 to 0.92], *p* = 0.04) with the same directional association (Table 2, Figs. 1 and 2).

**Table 2:** Linear mixed model results of MCI and Dementia groups for plasma TG/HDL ratios and ApoA1 levels, and CSF ApoA1 levels

	**MCI group**		**Dementia group**	
**Effect**	**Slope** **(95% CI)**	***p*-value**	**Slope** **(95% CI)**	***p*-value**
**MMSE**				
CSF ApoA1	0.204 (0.030,0.377)	***0.022***	− 0.683 (− 1.525,0.159)	0.11
Plasma ApoA1	− 0.498 (− 0.744, − 0.253)	**< *0.001***	− 1.546 (− 3.080, − 0.012)	***0.048***
TG/HDL ratio	− 0.127 (− 0.251, − 0.004)	***0.043***	− 0.299 (− 1.063, 0.466)	0.44
**CDR-SB**				
CSF ApoA1	− 0.025 (− 0.148, 0.099)	0.69	0.112 (− 0.383, 0.607)	0.65
Plasma ApoA1	0.141 (− 0.041, 0.324)	0.13	0.784 (− 0.118, 1.686)	0.088
TG/HDL ratio	0.116 (0.024, 0.207)	***0.013***	0.472 (0.023, 0.921)	***0.040***
**LM**				
CSF ApoA1	− 0.002 (− 0.144, 0.140)	0.97	0.203 (− 0.145, 0.550)	0.25
Plasma ApoA1	− 0.303 (− 0.502, − 0.103)	***0.003***	− 0.424 (− 1.053, 0.204)	0.18
TG/HDL ratio	0.009 (− 0.091,0.109)	0.87	0.141 (− 0.182, 0.464)	0.39

*MMSE*, Mini-Mental Status Exam score; *CDR-SB*, Clinical Dementia Rating Scale–Sum of Boxes score; *LM*, logical memory delayed recall score. Positive slope notes faster progression CDR-SB; negative slope notes faster progression in MMSE and LM

### Hypothesis 2: Plasma and CSF levels of ApoA1 and longitudinal cognitive decline

Among subjects in the MCI group, baseline plasma ApoA1 was associated with a decline in MMSE (β − 4.98 [95% CI, − 0.74 to − 0.25], *p* < 0.001) and LM scores (β − 0.303 [95% CI, − 0.50 to − 0.10], *p* = 0.003). Among both MCI and dementia groups, *higher* plasma ApoA1 had a faster rate of decline. Similarly, in the dementia group, baseline plasma ApoA1 was also associated with MMSE decline (β − 1.55 [95% CI –3.01 to − 0.01], *p* = 0.048) (Table 2, Figs. 1 and 2). Conversely, *lower* CSF ApoA1 level had a faster rate of decline in MMSE (β 0.20 [95% CI, 0.03 to 0.38], *p* = 0.022) among MCI subjects. Key results remained robust after multiple imputation and sensitivity analysis (Table 3).

**Table 3:** Sensitivity analysis of linear mixed model results for the MCI and dementia groups for plasma TG/HDL, ApoA1, and CSF ApoA1 using imputed data

	**MCI group**		**Dementia group**	
**Effect**	**Slope (95% CI)**	***p*-value**	**Slope (95% CI)**	***p*-value**
**MMSE**				
CSF ApoA1	0.219 (0.068, 0.370)	***0.005***	− 0.407 (− 1.157, 0.344)	0.29
Plasma ApoA1	− 0.492 (− 0.717, − 0.266)	** < *0.001***	− 1.207 (− 2.537, 0.123)	0.075
TG/HDL	− 0.165 (− 0.275, − 0.054)	***0.004***	− 0.247 (− 0.950, 0.455)	0.49
**CDR-SB**				
CSF ApoA1	− 0.065 (− 0.174, 0.044)	0.24	− 0.015 (− 0.446, 0.417)	0.95
Plasma ApoA1	0.173 (0.006, 0.340)	***0.043***	0.522 (− 0.246, 1.290)	0.18
TG/HDL	0.152 (0.070, 0.234)	** < *0.001***	0.402 (− 0.002, 0.807)	0.051
**LM**				
CSF ApoA1	0.039 (− 0.086, 0.165)	0.54	0.118 (− 0.176, 0.412)	0.43
Plasma ApoA1	− 0.280 (− 0.467, − 0.094)	***0.003***	− 0.344 (− 0.862, 0.174)	0.19
TG/HDL	− 0.010 (− 0.101, 0.082)	0.83	0.134 (− 0.147, 0.415)	0.35

Linear mixed effect models

*MMSE* Mini-Mental Status Exam score, *CDR-SB* Clinical Dementia Rating Scale–Sum of Boxes score, *LM* Logical memory delayed recall score

### Relationship between CSF, plasma ApoA1, and BBB biomarkers

**Fig. 3:**
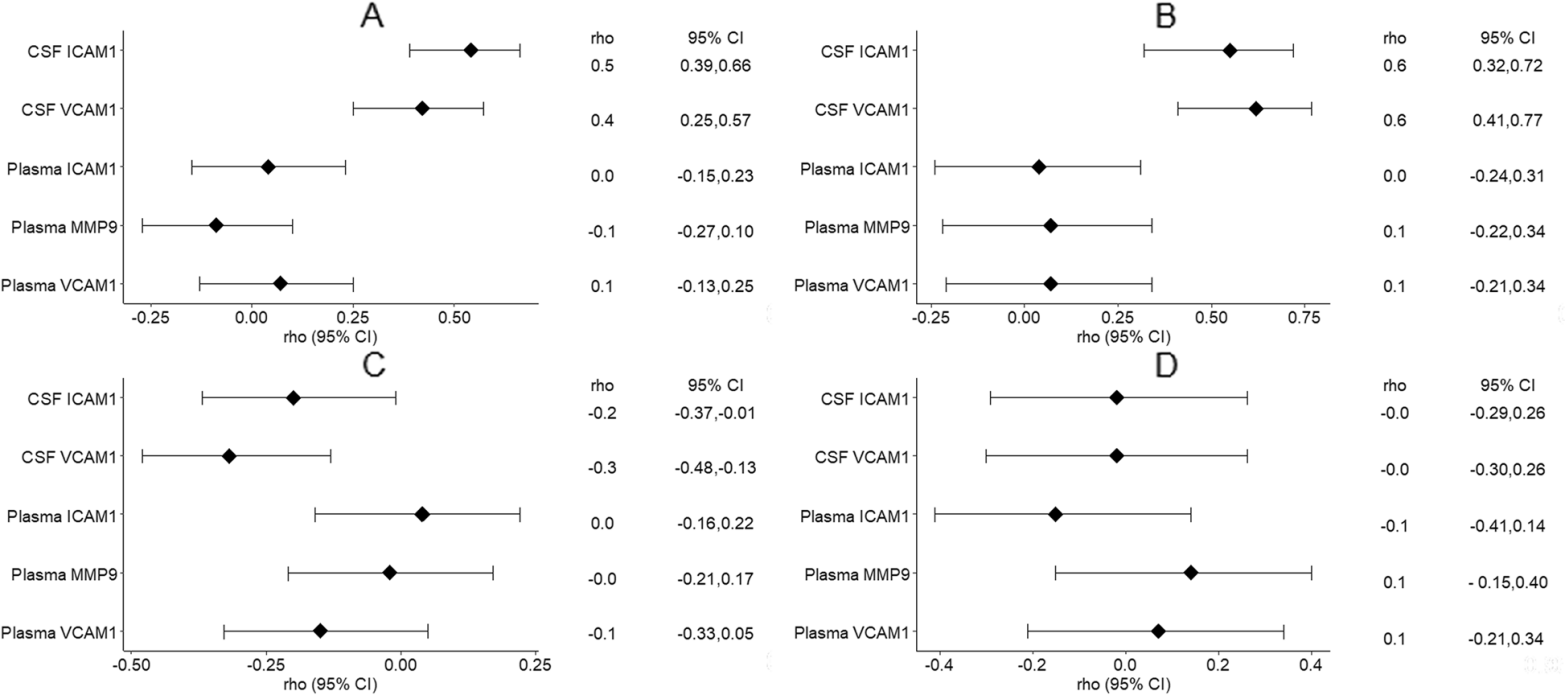
Pearson correlation forest plots. **A**. CSF ApoA1 BBB markers in MCI group, **B**. CSF ApoA1 BBB markers in dementia group, **C**. Plasma ApoA1 BBB markers in MCI group, **D**. Plasma ApoA1 BBB markers in the dementia group

In the MCI group, CSF ApoA1 levels correlated positively with CSF ICAM1 and VACM1 levels [correlation range 0.42 to 0.54, all *p* < 0.001], while plasma ApoA1 levels correlated negatively with CSF ICAM1 and VCAM1 levels [correlation range − 0.20 to − 0.34, *p* < 0.001 to 0.04] (Tables 4 and 5, Fig. 3).

**Table 4:** Correlations between lipid variables and CSF and plasma BBB integrity biomarkers in the MCI group

		**All MCI**			**Apoeε4 + **			**Apoe4ε -**		
**Factor 1**	**Factor 2**	***N***	**rho (95% CI)**	***p*-value**	***N***	**rho (95% CI)**	***p*-value**	***N***	**rho (95% CI)**	***p*-value**
**CSF ApoA1**	**CSF ICAM1**	106	0.54 (0.39, 0.66)	** < *0.001***	58	0.51 (0.29, 0.68)	** < *0.001***	48	0.59 (0.37, 0.75)	** < *0.001***
	**CSF VCAM1**	106	0.42 (0.25, 0.57)	** < *0.001***	58	0.31 (0.06, 0.53)	***0.017***	48	0.50 (0.25, 0.69)	** < *0.001***
	**Plasma ICAM1**	106	0.04 (− 0.15, 0.23)	0.69	58	0.03 (− 0.23, 0.29)	0.81	48	− 0.00 (− 0.29, 0.28)	0.99
	**Plasma MMP9**	106	− 0.09 (− 0.27, 0.10)	0.37	58	− 0.11 (− 0.36, 0.16)	0.43	48	− 0.05 (− 0.33, 0.23)	0.72
	**Plasma VCAM1**	106	0.07 (− 0.13, 0.25)	0.50	58	0.15 (− 0.12, 0.39)	0.27	48	− 0.07 (− 0.35, 0.22)	0.62
**Plasma ApoA1**	**CSF ICAM1**	106	− 0.20 (− 0.37, − 0.01)	*0.044*	58	− 0.18 (− 0.42, 0.08)	0.18	48	− 0.22 (− 0.47, 0.07)	0.13
	**CSF VCAM1**	106	− 0.32 (− 0.48, − 0.13)	** < *0.001***	58	− 0.44 (− 0.62, − 0.20)	** < *0.001***	48	− 0.18 (− 0.44, 0.11)	0.22
	**Plasma ICAM1**	106	0.04 (− 0.16, 0.22)	0.72	58	0.21 (− 0.05, 0.44)	0.12	48	− 0.11 (− 0.38, 0.18)	0.48
	**Plasma MMP9**	106	− 0.02 (− 0.21, 0.17)	0.86	58	− 0.16 (− 0.40, 0.10)	0.22	48	0.15 (− 0.14, 0.42)	0.30
	**Plasma VCAM1**	106	− 0.15 (− 0.33, 0.05)	0.13	58	− 0.07 (− 0.33, 0.19)	0.58	48	− 0.16 (− 0.42, 0.13)	0.28
**TG/HDL**	**CSF ICAM1**	106	0.09 (− 0.11, 0.27)	0.38	58	− 0.06 (− 0.31, 0.20)	0.68	48	0.25 (− 0.04, 0.50)	0.092
	**CSF VCAM1**	106	0.32 (0.14, 0.48)	** < *0.001***	58	0.34 (0.09, 0.55)	***0.009***	48	0.30 (0.02, 0.54)	*0.038*
	**Plasma ICAM1**	106	0.01 (− 0.18, 0.20)	0.92	58	− 0.14 (− 0.39, 0.12)	0.29	48	0.19 (− 0.10, 0.45)	0.20
	**Plasma MMP9**	106	0.13 (− 0.06, 0.31)	0.19	58	0.21 (− 0.05, 0.44)	0.11	48	0.02 (− 0.27, 0.30)	0.90
	**Plasma VCAM1**	106	0.12 (− 0.07, 0.31)	0.21	58	− 0.12 (− 0.36, 0.15)	0.39	48	0.36 (0.08, 0.58)	*0.012*

*rho* Pearson’s correlation, *CI* Confidence interval, *FDR* < 0.05 in bold, *ApoA1* Apolipoprotein, *TG/HDL* Plasma triglyceride/HDL cholesterol ratio

**Table 5:** Correlations between lipid variables and CSF and plasma BBB integrity biomarkers in the dementia group

		**All dementia**			**Apoe4 ε + **			**Apoe4ε-**		
**Factor 1**	**Factor 2**	***N***	**rho (95% CI)**	***p*-value**	***N***	**rho (95% CI)**	***p*-value**	***N***	**rho (95% CI)**	***p*-value**
**CSF ApoA1**	**CSF ICAM1**	50	0.55 (0.32, 0.72)	** < *0.001***	36	0.64 (0.40, 0.80)	** < *0.001***	14	0.39 (− 0.18, 0.76)	0.18
	**CSF VCAM1**	50	0.62 (0.41, 0.77)	** < *0.001***	36	0.64 (0.40, 0.80)	** < *0.001***	14	0.57 (0.06, 0.85)	*0.030*
	**Plasma ICAM1**	50	0.04 (− 0.24, 0.31)	0.79	36	0.18 (− 0.16, 0.48)	0.29	14	− 0.31 (− 0.72, 0.27)	0.30
	**Plasma MMP9**	50	0.07 (− 0.22, 0.34)	0.65	36	− 0.02 (− 0.35, 0.31)	0.90	14	0.29 (− 0.29, 0.71)	0.33
	**Plasma VCAM1**	50	0.07 (− 0.21, 0.34)	0.63	36	0.06 (− 0.27, 0.38)	0.73	14	0.09 (− 0.46, 0.59)	0.76
**Plasma ApoA1**	**CSF ICAM1**	50	− 0.02 (− 0.29, 0.26)	0.90	36	− 0.02 (− 0.34, 0.31)	0.93	14	− 0.02 (− 0.55, 0.52)	0.94
	**CSF VCAM1**	50	− 0.02 (− 0.30, 0.26)	0.89	36	0.02 (− 0.31, 0.35)	0.89	14	− 0.19 (− 0.65, 0.38)	0.52
	**Plasma ICAM1**	50	− 0.15 (− 0.41, 0.14)	0.31	36	− 0.14 (− 0.45, 0.20)	0.41	14	− 0.19 (− 0.65, 0.38)	0.52
	**Plasma MMP9**	50	0.14 (− 0.15, 0.40)	0.34	36	0.14 (− 0.20, 0.45)	0.42	14	0.11 (− 0.44, 0.61)	0.70
	**Plasma VCAM1**	50	0.07 (− 0.21, 0.34)	0.63	36	0.15 (− 0.19, 0.46)	0.38	14	− 0.20 (− 0.66, 0.37)	0.50
**TG/HDL**	**CSF ICAM1**	50	− 0.00 (− 0.28, 0.28)	0.98	36	− 0.07 (− 0.39, 0.26)	0.67	14	0.11 (− 0.45, 0.61)	0.71
	**CSF VCAM1**	50	− 0.09 (− 0.36, 0.19)	0.51	36	− 0.17 (− 0.47, 0.17)	0.33	14	0.06 (− 0.49, 0.57)	0.85
	**Plasma ICAM1**	50	0.30 (0.02, 0.53)	*0.036*	36	0.28 (− 0.05, 0.56)	0.097	14	0.34 (− 0.23, 0.74)	0.24
	**Plasma MMP9**	50	− 0.20 (− 0.45, 0.09)	0.18	36	− 0.10 (− 0.41, 0.24)	0.57	14	− 0.43 (− 0.78, 0.13)	0.13
	**Plasma VCAM1**	50	0.01 (− 0.27, 0.29)	0.95	36	− 0.13 (− 0.44, 0.20)	0.44	14	0.39 (− 0.18, 0.76)	0.17

*rho*, Pearson’s correlation; *CI*, confidence interval; *FDR* < 0.05 in bold; *ApoA1*, apolipoprotein; *TG/HDL*, plasma triglyceride/HDL cholesterol ratio

In the dementia group, CSF ApoA1 levels correlated positively with CSF ICAM1 and VACM1 levels [correlation range 0.55 to 0.62, all *p* < 0.001], while a negative correlation was observed between plasma ApoA1 and CSF ICAM1 and VCAM1, but this correlation was not significant.

### Stratification by APOE ε4 status

To further evaluate the differential effects of *APOE ε4* on key plasma and CSF analytes, we evaluated the robustness of above results among smaller sample sizes of *APOE ε4* carriers and non-carriers.

Among both *APOE ε4* carriers and non-carriers in the MCI and Dementia groups, CSF ApoA1 levels correlated positively with CSF ICAM1 and VCAM1 levels [correlation range 0.42 to 0.59, all *p* < 0.001], while plasma ApoA1 levels correlated negatively with CSF ICAM1 (− 0.20, *p* = 0.04) and VCAM1 levels (− 0.32. *p* < 0.001) only among MCI group *APOE ε4*carriers (Table 6).

**Table 6:** Summary of Linear mixed model results of MCI and Dementia groups and by *APOE ε4* status for plasma TG/HDL ratio and ApoA1, and CSF ApoA1 levels

Analyte	All MCI (*n* = 106)	MCI APOE4 positive (*n* = 58)	MCI APOE4 negative (*n* = 48)	All dementia (*n* = 50)	Dementia APOE4 positive (*n* = 36)	Dementia APOE4 negative (*n* = 14)
Plasma TG/HDL	Faster	Faster	N.S	Faster	Faster	N.S
Plasma ApoA1	Faster	Faster	Faster	Faster	Faster	Faster
CSF ApoA1	Slower	Faster	N.S	N.S	Faster	N.S

Faster: Higher baseline analyte levels relate to faster progression in one or more of the three cognitive scores: MMSE, CDR-SB, LM

Slower: Higher baseline analyte levels relate to slower progression in one or more of the three cognitive scores: MMSE, CDR-SB, LM

*N.S* Non-significant results

Among the MCI *APOE ε4* carriers, higher plasma TG/HDL-C had a faster rate of decline in MMSE and CDR-SB. Among both MCI *APOE ε4* non-carriers and carriers, higher plasma ApoA1 had a faster rate of decline in MMSE (Tables 6 and 7). Higher plasma ApoA1 in MCI *APOE ε4* non-carriers alone had a faster rate of decline in LM scores (β − 0.60 [95% CI, − 0.87 to − 0.33], *p* < 0.001). Higher CSF ApoA1 in MCI *APOE ε4* carriers also had a faster rate of decline in CDR-SB (*β* 0.22 [95% CI, 0.06 to 0.38], *p* = 0.008).

**Table 7:** Linear mixed model results of the MCI and dementia groups stratified by *APOE* ε4 status

	**MCI group**				**Dementia group**			
	**APOEε4 positive (*n* = 58)**		**APOEε4 negative (*n* = 48)**		**APOEε4 positive (*n* = 36)**		**APOEε4 negative (*n* = 14)**	
**Effect**	**Slope (95% CI)**	***p*-value**	**Slope (95% CI)**	***p*-value**	**Slope (95% CI)**	***p*-value**	**Slope (95% CI)**	***p*-value**
**MMSE**								
CSF ApoA1	− 0.059 (0.302, 0.183)	0.63	0.233 (− 0.024, 0.489)	0.075	− 1.029 (− 1.950, − 0.108)	**0.029**	1.078 (− 0.205, 2.361)	0.097
Plasma ApoA1	− 0.798 (− 1.258, − 0.339)	** < 0.001**	− 0.474 (− 0.740, − 0.209)	** < *0.001***	− 1.715 (− 3.285, − 0.146)	**0.033**	− 0.794 (− 3.923, 2.336)	0.61
Plasma TG/HDL	− 0.327 (− 0.562, − 0.093)	**0.006**	− 0.044 (− 0.173, 0.084)	0.50	− 0.559 (− 1.279, 0.162)	0.13	0.960 (− 1.045, 2.965)	0.34
**CDR-SB**								
CSF ApoA1	0.220 (0.057, 0.383)	**0.008**	0.076 (− 0.113, 0.264)	0.43	0.137 (− 0.451, 0.725)	0.64	− 0.474 (− 1.245, 0.297)	0.22
Plasma ApoA1	0.518 (0.199, 0.838)	**0.002**	− 0.032 (− 0.229, 0.164)	0.75	0.656 (− 0.346, 1.658)	0.19	0.729 (− 1.151, 2.609)	0.44
TG/HDL	0.287 (0.126, 0.448)	** < 0.001**	0.057 (− 0.038, 0.152)	0.24	0.580 (0.120, 1.040)	**0.014**	− 0.112 (− 1.316, 1.093)	0.85
**LM**								
CSF ApoA1	0.017 (− 0.156, 0.190)	0.84	0.093 (− 0.163, 0.350)	0.47	0.042 (− 0.282, 0.366)	0.79	0.613 (− 0.008, 1.234)	0.053
Plasma ApoA1	0.220 (− 0.108, 0.549)	0.19	− 0.598 (− 0.866, − 0.330)	** < *0.001***	0.068 (− 0.479, 0.615)	0.80	− 2.753 (− 4.268, − 1.239)	**< *0.001***
Plasma TG/HDL	0.125 (− 0.044, 0.293)	0.15	0.002 (− 0.127, 0.130)	0.98	0.142 (− 0.117, 0.402)	0.28	− 0.548 (− 1.518, 0.423)	0.26

*MMSE* Mini-Mental Status Exam score, *CDR-SB* Clinical Dementia Rating Scale–Sum of Boxes score, *LM* Logical memory delayed recall score. Positive slope notes faster progression CDR-SB, negative slope notes faster progression in MMSE and LM

Among dementia *APOE ε4* carriers, higher plasma TG/HDL-C had a faster rate of decline in CDR-SB and higher plasma ApoA1 had a faster rate of decline in MMSE. Higher plasma ApoA1 in dementia *APOE ε4* non-carriers also related to faster decline in LM scores (*β* − 2.75 [95% CI, − 4.27 to − 1.24], *p* < 0.001). Among dementia *APOE ε4* carriers, higher CSF ApoA1 also had a faster rate of decline in MMSE (*β* − 1.03 [95% CI, − 1.95 to − 0.11], *p* = 0.029).

### Exploratory inflammatory analyte analysis

In MCI patients, after FDR correction, (a) plasma TG/HDL ratio was positively correlated to CSF (CRP, VCAM1) and plasma (C3, Fibrinogen, ferritin, IL-16) and negatively correlated to plasma (IL-13, IL-3), (b) plasma ApoA1 negatively correlated to CSF (AAT, CRP, VCAM1) and plasma (B2M, Ferritin) and positively correlated to plasma (IL-13, IL-3), and (c) CSF ApoA1 positively correlated to CSF (A2M, AAT, B2M, CRP, CCL2, ICAM1, IL-16, IL-3, IL-6r, IL-8, MMIF, PAI1, TIMP1, VCAM1, VEGF, vWF) but CSF ApoA1 did not correlate to plasma inflammation markers measured (Supplementary table 1).

In dementia patients, after FDR correction, (a) both plasma TG/HDL ratio and plasma ApoA1 were not correlated to CSF inflammatory analytes but negatively correlated to plasma (IL-3), but plasma TG/HDL positively correlated to plasma (C3, CCL11) and (b) CSF ApoA1 positively correlated to CSF (A2M, AAT, B2M, CRP, ICAM1, IL-16, IL-3, IL-8, MMP2, PAI1, TIMP1, VCAM1, VEGF, vWF) and negatively correlated to plasma (IL-3) (Supplementary table 2).

Among both MCI and dementia groups, the top hit among the Kyoto Encyclopedia of Genes and Genomes (KEGG) pathways [36] for CSFApoA1 (analyte count 7,* P* < 0.0001) was the advanced glycation end products(AGE)-receptors for AGE (RAGE) signaling pathway in diabetic complications (KEGG hsa04933). There were not enough inflammatory analytes correlated to TG/HDL and plasma APOA1 for pathway analysis.

## Discussion

This study examined longitudinal cognitive outcomes in 156 MCI and AD dementia participants from the ADNI cohort. Our results indicate that elevated biomarkers of metabolic syndrome, higher plasma TG/HDL-C ratios, and plasma ApoA1 levels had a faster cognitive and functional rate of decline in both MCI and AD dementia participants. The results for plasma ApoA1 were significant for both *APOE ε4* carriers and non-carriers in the MCI and dementia groups for at least one of the three cognitive measures tested (MMSE, CDR-SB, and LM). Notably, CSF and plasma ApoA1 levels show opposite directional correlations with CSF biomarkers of BBB integrity and inflammation markers. CSF ApoA1, but not plasma ApoA1, positively correlates with CSF VCAM1 and CSF ICAM1, regardless of *APOE ε4* status and inflammation markers of AGE-RAGE signaling pathway in diabetic complications. We have also previously reported that plasma ApoA1 is positively correlated with CSF t-tau and p-tau, while CSF ApoA1 is negatively correlated with both biomarkers [32]. These results together suggest that plasma and CNS ApoA1 levels are likely differentially modulated in relation to BBB and inflammatory biomarker changes. The clinical implication of this physiological difference between the plasma and CNS ApoA1 pools was noted in the MCI group, where *higher* plasma ApoA1 was associated with rapid decline in MMSE and LM scores, whereas *lower* CSF ApoA1 was associated with rapid decline in MMSE scores.

High plasma TG/HDL-C ratios are related to metabolic syndrome [6] and insulin resistance [9]. Insulin resistance can alter systemic lipid metabolism and induce endothelial dysfunction, which together contributes to atherosclerotic plaque formation, altered signal transduction, and delivery of substrates to the myocardium, as noted in coronary disease [7, 37]. Low TG/HDL ratios have been associated with higher scores in memory performance among elderly subjects (> 80 years) [38]. Plasma levels of TG and HDL-C have not been shown to significantly differ between AD dementia, MCI, and healthy controls in a recent meta-analysis [39], which is consistent with the results of the TG/HDL ratio in the current study. Among normal cognition subjects, TG levels correlate with longitudinal cognitive outcomes differently based on the age group examined [40], while high HDL-C levels have been associated with a significantly decreased risk of AD, with a few exceptions [41]. However, no prior studies have evaluated the association of the TG/HDL-C ratio with the longitudinal rate of cognitive and functional decline in MCI and AD dementia, as was performed in the current study. Among MCI subjects in this study, a 4 × difference in the TG/HDL-C ratio relates to a 1/2 point faster progression in MMSE score per year.

Endothelial BBB changes have been noted in AD-related cognitive deficits [42]. Soluble CSF VCAM1 and ICAM1 levels, which reflect changes in the endothelial layer of the BBB, have been correlated with CNS inflammatory changes [43], while in the periphery, VCAM1, and ICAM1 are upregulated in hypercholesterolemia and associated with early atherogenic lesions [44]. Among CNS diseases, CSF levels of VCAM1 and ICAM1 have been noted to correlate more strongly with disease-related outcomes than plasma levels [45, 46]. Taken together, the CSF and plasma levels of ICAM1 and VCAM1 may reflect different pathophysiological processes, with CSF levels of VCAM1 and ICAM1 suggesting BBB-related endothelial damage, and their plasma levels noting peripheral lipidogenic metabolism and atherogenic changes.

In the periphery, ApoA1 is also reported to modulate intestinal homeostasis and microbiota composition, and ApoA1 deficiency-driven dysbiosis may contribute to inflammation or predispose to atherosclerosis development [47], which supports potentially different pathophysiological roles for plasma and CNS ApoA1 pools in AD dementia. Consistent with this, in the current study, unlike the positive correlations between CSF ApoA1 and CSF BBB and inflammation biomarkers, we did not observe significant positive correlations between plasma ApoA1 and CSF BBB and inflammation biomarkers. This would suggest that plasma ApoA1 impact on cognitive outcomes is less likely related to CNS inflammatory and endothelial changes, in contrast to CSF ApoA1.

In AD animal models, over-expression of ApoA1 has been noted to slow the development of age-related learning and memory deficits, despite continued amyloid β deposition, and to associate with decreased neuroinflammation [20]. ApoA1 prevents the formation of Aβ42 aggregates and decreases Aβ42 toxicity in primary brain cells [19]. Consistent with these findings, our results show that higher CSF ApoA1 is related to a slower cognitive decline among the MCI participants.

Analysis of MCI and dementia group cognitive outcomes by *APOE ε4* status revealed that higher CSF ApoA1 levels in *APOE ε4* carriers had a more rapid clinical progression. This analysis was necessarily limited by sample size, so these results require additional research. However, similar findings among *APOE* ε4 carriers with subjective cognitive impairment and higher CSF ApoA1 have been associated with an increased risk of clinical progression toward AD [26].

One possibility for the *lower* CSF ApoA1 in the whole MCI group being associated with the rapid decline in MMSE, whereas a *higher* CSF ApoA1 among *APOE ε4* carriers related to a more rapid clinical progression could be that there is a compensatory upregulation in CSF ApoA1, as a protective factor in the face of enhanced neurodegeneration or cellular stressors, as is noted for some other cell-protective inflammatory analytes [48]. The positive correlation noted in this study between CSF ApoA1 and CSF inflammation markers of AGE-RAGE signaling pathway in diabetes is also consistent with this. If validated, the association of CSF ApoA1 with rapid clinical progression among *APOE ε4* carriers and its positive correlation with inflammation biomarkers of AGE-RAGE signaling would not be directly causal.

In both MCI and dementia stages of AD, the levels of plasma ApoA1 are decreased compared to cognitive normal subjects in most studies, with few exceptions, while CSF ApoA1 is reported as unchanged or decreased between disease stages across different studies [16]. The MCI group in the current study had higher plasma ApoA1 levels than the Dementia group. In both MCI and dementia groups, higher plasma ApoA1 was associated with faster progression in MMSE and CDR-SB. In the Amsterdam Dementia Cohort, no significant associations between either CSF or plasma ApoA1 and cognitive decline were found among MCI participants, but the trend was in a similar direction [26]. In categorical analyses limited to normal cognition elders converting to dementia over 2 years in the Sydney Memory and Ageing study, lower plasma ApoA1 levels were associated with an increased risk of dementia [49]. One prospect that has been raised to explain these discrepant results in ApoA1 is that ApoA1, like other neuroinflammatory analytes (sTREM2 and sTNFR2) [48], could increase in the early preclinical phase of AD with an increasing rate of neuropathology burden. Yet as the disease progresses to the clinical symptomatic phase of AD, there could be a decrease in ApoA1 levels when neuropathology levels are not rapidly changing, resulting in differing clinical and imaging outcomes for ApoA1 levels depending on the stage of AD studied [14, 26]. In addition, the frequency of medical co-morbidities including differences in insulin resistance, obesity, lipid profile-modifying medications, racial differences, and relative numbers of *APOE* ε4 carriers may impact differences in results between cohorts. Methodological differences across studies (categorical outcomes versus continuous outcomes as in the current study) could be another reason for differences between studies. Differences in ApoA1 effects between studies could also be a harbinger suggesting that any future therapeutic benefit of modifying ApoA1 levels and the TG/HDL-C ratio to improve cognitive outcomes could differ based on the specific population targeted: normal cognition elderly, MCI, and AD dementia stages and, likely, between other subpopulations, e.g., based on insulin resistance or *APOE* ε4 status. Targeting ApoA1 levels and the TG/HDL-C ratio by diet and/or medication options in these clinical subjects warrants future studies.

## Limitations

The current study is unique in evaluating plasma TG/HDL-C ratio and both CSF and plasma levels of ApoA1, along with key plasma and CSF BBB and inflammation biomarkers, to clarify the potential pathophysiological role of TG/HDL-C and ApoA1 in the CNS versus periphery in MCI and AD dementia stages. Additional evaluation of cognitive normal subjects and preclinical AD is needed to better understand the implications of these results across all stages of AD. However, this analysis could not be meaningfully expanded to include cognitively normal subjects in the ADNI cohort due to the small size of analysis groups when categorized by their amyloid- and tau-positive biomarkers and APOA1 data. The current results are among a well-characterized cohort with AD biomarkers, and key metabolic syndrome related to medical history. The analysis also accounted for BMI and the use of lipid-modifying medications; however, the effect of other medication interactions and diet on lipid metabolism on the results cannot be entirely ruled out. Another limitation is that the effects of exercise, co-existing inflammatory diseases, biomarkers of diabetes, and cardiovascular risk could not be evaluated in the current study. These interacting factors are worthy of further study. Although the statistical significance level of *p* < 0.05 is modest, additional sensitivity analyses corroborate the robustness of these effects.

The current results are likely generalizable to MCI and AD dementia subjects without severe metabolic syndrome in a longitudinal clinical cohort. However, given that the ADNI cohort is predominantly White with a higher level of education, there is still a need for replication studies of these results among other racial and ethnic cohorts with diverse educational and socioeconomic backgrounds. In addition, subjects with mixed dementia pathology and concomitant vascular comorbidities constitute a significant proportion of dementia subjects in the community [50] and the significance of these results among them is unclear.

## Conclusions

Biomarkers of metabolic syndrome relate to rate of cognitive decline even after controlling for BMI in MCI and dementia individuals. CSF and plasma levels of ApoA1 differentially associate with clinical outcomes in MCI and AD dementia. Plasma levels of ApoA1 and the TG/HDL-C ratio appear to be promising prognostic markers for the rate of cognitive decline in MCI and AD dementia subjects and their therapeutic modification by diet and medication options in these clinical subjects is likely to be of high future interest. These results need additional replication in multiethnic cohorts.

## Supplementary Information

**Additional file 1.** Supplementary material

**Additional file 2:** **Supplementary Table 1.** Associations between TG/HDL, APOA1 and AD and inflammatory biomarkers in MCI patients

**Additional file 3:** **Supplementary Table 2.** Associations between TG/HDL, APOA1 and AD and inflammatory biomarkers in Dementia patients

## Data Availability

The ADNI data analyzed are available in the ADNI repository, http://adni.loni.usc.edu/.

## Abbreviations

AD: Alzheimer’s disease
ADNI: Alzheimer's Disease Neuroimaging Initiative
ApoA1: Apolipoprotein A1
BBB: Blood-brain barrier
BMI: Body mass index
CSF: Cerebrospinal fluid
CNS: Central nervous system
CDR-SB: Clinical Dementia Rating–Sum of Boxes
FDR: False discovery rate
HDL: High-density lipoproteins
ICAM: Intercellular Adhesion Molecule 1
LM: Logical memory delayed recall
MMP9: Matrix metallopeptidase 9
MMSE: Mini-Mental State Exam
MCI: Mild cognitive Impairment
TG: Plasma triglyceride
TG/HDL-C: Plasma triglyceride-to-HDL cholesterol
VCAM1: Vascular cell adhesion protein 1
