# Supplementary material for: Metabolic syndrome biomarkers relate to rate of cognitive decline in MCI and dementia stages of Alzheimer’s disease
Source: Alzheimers Res Ther. 2023 Mar 16;15:54. doi: 10.1186/s13195-023-01203-y (PMC10018847; doi:10.1186/s13195-023-01203-y)
Supplement: Supplementary file 1 — Additional file 1. Supplementary material [file 13195_2023_1203_MOESM1_ESM.docx]

1. **List of lipids modifying medications evaluated in the cohort**
2. Alpha Lipoic Acid
3. Atorvastatin
4. Cholestoff
5. Cholestyramine
6. Ezetimibe
7. Ezetimibe and simvastatin
8. Fenofibrate
9. Flax seed oil
10. Fluvastatin
11. Gemfibrozil
12. Lovastatin
13. Niacin
14. Niacinamide
15. Parvastatin
16. Rosuvastatin
17. Simvastatin

**2. List of Inflammatory analytes evaluated**

| **RBM Name** | **Gene** | **RBM Name** | **Gene** |
| --- | --- | --- | --- |
| 1.      Alpha-1-Antitrypsin | AAT | 29.  Interleukin-12 Subunit p40 | IL12B |
| 2.      Alpha-2-Macroglobulin | A2M | 30.  Interleukin-12 Subunit p70 | IL12P70 |
| 3.      Beta-2-Microglobulin | B2M | 31.  Interleukin-15 | IL15 |
| 4.      Brain-Derived Neurotrophic Factor | BDNF | 32.  Interleukin-17 | IL17A |
| 5.      Complement C3 | C3 | 33.  Interleukin-18 | IL18 |
| 6.      C-Reactive Protein | CRP | 34.  Interleukin-8 | CXCL8 |
| 7.      Eotaxin-1 | CCL11 | 35.  Interleukin-23 | IL23A |
| 8.      Fibrinogen | FGA | 36.  Macrophage Inflammatory Protein-1 alpha | CCL3 |
| 9.  Factor VII | F7 | 37.  Macrophage Inflammatory Protein-1 beta | CCL4 |
| 10.  Ferritin | FTH1 | 38.Macrophage migration inhibitory factor | MMIF |
| 11.  Granulocyte-Macrophage Colony-Stimulating Factor | CSF2 | 39.  Matrix Metalloproteinase-3 | MMP3 |
| 12.  Granulocyte Colony-Stimulating Factor | CSF3 | 40.  Matrix Metalloproteinase-9 | MMP9 |
| 13.  Haptoglobin | HP | 41.  Monocyte Chemotactic Protein 1 | CCL2 |
| 14.  Intercellular Adhesion Molecule 1 | ICAM1 | 42.  Matrix Metalloproteinase-2 | MMP2 |
| 15.  Interferon gamma | IFNG | 43.  Myeloperoxidase | MPO |
| 16.  Interleukin-1 alpha | IL1A | 44.  Neuron-Specific Enolase | ENO2 |
| 17.  Interleukin-1 beta | IL1B | 45.  Plasminogen Activator Inhibitor 1 | SERPINE1 |
| 18.  Interleukin-1 receptor antagonist | IL1RN | 46.  Serotransferrin | TF |
| 19.  Interleukin-2 | IL2 | 47.  Stem Cell Factor | SCF |
| 20.  Interleukin-3 | IL3 | 48.  T-Cell-Specific Protein RANTES | CCL5 |
| 21.  Interleukin-4 | IL4 | 49.  Tissue Inhibitor of Metalloproteinases 1 | TIMP1 |
| 22.  Interleukin-5 | IL5 | 50.  Tumor Necrosis Factor alpha | TNF |
| 23.  Interleukin-6r | IL6r | 41.  Tumor Necrosis Factor beta | LTA |
| 24.  Interleukin-7 | IL7 | 42.  Tumor necrosis Factor Receptor 2 | TNFRSF1B |
| 25.  Interleukin-10 | IL10 | 53.  Vascular Cell Adhesion Molecule-1 | VCAM1 |
| 26.  Interleukin-12 Subunit p40 | IL12B | 54.  Vascular Endothelial Growth Factor | VEGFA |
| 27.  Interleukin-12 Subunit p70 | IL12P70 | 55.  Vitamin D-Binding Protein | GC |
| 28.  Interleukin-15 | IL15 | 56.  von Willebrand Factor | VWF |
