# Supplementary material for: Metabolic syndrome biomarkers relate to rate of cognitive decline in MCI and dementia stages of Alzheimer’s disease
Source: Alzheimers Res Ther. 2023 Mar 16;15:54. doi: 10.1186/s13195-023-01203-y (PMC10018847; doi:10.1186/s13195-023-01203-y)
Supplement: Supplementary file 2 — Additional file 2: Supplementary Table 1. Associations between TG/HDL, APOA1 and AD and inflammatory biomarkers in MCI patients [file 13195_2023_1203_MOESM2_ESM.docx]

| **Supplementary Table 1. Associations between TG/HDL, APOA1 and AD and inflammatory biomarkers in MCI patients** | | | | | | | |
| --- | --- | --- | --- | --- | --- | --- | --- |
|  |  | **Plasma TG/HDL** | | **CSF ApoAI** | | **Plasma ApoAI** | |
| **Factor** | **N** | **rho (95% CI)** | **p-value** | **rho (95% CI)** | **p-value** | **rho (95% CI)** | **p-value** |
| **CSF Markers** |  |  |  |  |  |  |  |
| A-Beta42 | 106 | -0.01 (-0.20, 0.18) | 0.92 | 0.36 (0.18, 0.51) | ***<0.001*** | -0.14 (-0.32, 0.05) | 0.26 |
| P-Tau | 106 | -0.07 (-0.26, 0.12) | 0.65 | -0.05 (-0.24, 0.14) | 0.78 | 0.25 (0.06, 0.42) | 0.056 |
| Tau | 106 | -0.07 (-0.25, 0.13) | 0.67 | -0.02 (-0.21, 0.17) | 0.92 | 0.23 (0.04, 0.40) | 0.072 |
| A2Macro | 106 | 0.08 (-0.11, 0.27) | 0.57 | 0.75 (0.65, 0.82) | ***<0.001*** | -0.18 (-0.35, 0.02) | 0.17 |
| AAT | 106 | 0.24 (0.05, 0.41) | 0.059 | 0.43 (0.26, 0.57) | ***<0.001*** | -0.35 (-0.51, -0.17) | ***0.003*** |
| ApoAI | 106 | -0.02 (-0.21, 0.17) | 0.90 | NA |  | -0.01 (-0.20, 0.18) | 0.95 |
| B2M | 106 | 0.14 (-0.05, 0.32) | 0.37 | 0.34 (0.15, 0.49) | ***0.002*** | -0.16 (-0.34, 0.03) | 0.19 |
| C3 | 106 | 0.15 (-0.04, 0.33) | 0.36 | 0.18 (-0.02, 0.35) | 0.21 | -0.27 (-0.44, -0.08) | 0.039 |
| CRP | 106 | 0.29 (0.10, 0.45) | ***0.019*** | 0.82 (0.75, 0.88) | ***<0.001*** | -0.37 (-0.52, -0.19) | ***0.003*** |
| ICAM1 | 106 | 0.09 (-0.11, 0.27) | 0.56 | 0.54 (0.39, 0.66) | ***<0.001*** | -0.20 (-0.37, -0.01) | 0.12 |
| IL16 | 106 | 0.16 (-0.03, 0.34) | 0.29 | 0.54 (0.39, 0.66) | ***<0.001*** | -0.22 (-0.39, -0.03) | 0.089 |
| IL25 | 106 | 0.06 (-0.14, 0.24) | 0.73 | 0.10 (-0.10, 0.28) | 0.56 | -0.10 (-0.28, 0.10) | 0.47 |
| IL3 | 106 | -0.02 (-0.21, 0.18) | 0.92 | 0.52 (0.37, 0.65) | ***<0.001*** | -0.11 (-0.29, 0.09) | 0.43 |
| IL6r | 106 | 0.11 (-0.08, 0.29) | 0.50 | 0.41 (0.24, 0.56) | ***<0.001*** | 0.01 (-0.18, 0.20) | 0.96 |
| IL8 | 106 | 0.02 (-0.17, 0.21) | 0.90 | 0.47 (0.31, 0.61) | ***<0.001*** | -0.16 (-0.34, 0.03) | 0.19 |
| MCP1 | 106 | 0.25 (0.06, 0.42) | 0.050 | 0.30 (0.11, 0.46) | ***0.006*** | -0.17 (-0.35, 0.02) | 0.19 |
| MCP2 | 106 | 0.05 (-0.14, 0.24) | 0.74 | -0.02 (-0.21, 0.17) | 0.90 | 0.01 (-0.18, 0.20) | 0.95 |
| MIP1B | 106 | 0.14 (-0.06, 0.32) | 0.37 | 0.16 (-0.03, 0.34) | 0.27 | -0.15 (-0.33, 0.04) | 0.23 |
| MMIP | 106 | 0.02 (-0.17, 0.21) | 0.90 | 0.33 (0.15, 0.49) | ***0.002*** | -0.00 (-0.19, 0.19) | 0.99 |
| MMP2 | 106 | 0.09 (-0.10, 0.27) | 0.56 | 0.02 (-0.18, 0.21) | 0.92 | -0.25 (-0.42, -0.06) | 0.056 |
| MMP3 | 106 | 0.01 (-0.18, 0.20) | 0.92 | 0.12 (-0.07, 0.30) | 0.47 | -0.01 (-0.20, 0.18) | 0.96 |
| PAI1 | 106 | 0.12 (-0.08, 0.30) | 0.45 | 0.24 (0.05, 0.41) | ***0.041*** | -0.20 (-0.37, -0.00) | 0.12 |
| SCF | 106 | 0.19 (-0.00, 0.37) | 0.19 | 0.21 (0.02, 0.38) | 0.11 | -0.24 (-0.41, -0.05) | 0.060 |
| TIMP1 | 106 | 0.11 (-0.09, 0.29) | 0.51 | 0.47 (0.31, 0.61) | ***<0.001*** | -0.21 (-0.39, -0.02) | 0.098 |
| VCAM1 | 106 | 0.32 (0.14, 0.48) | ***0.008*** | 0.42 (0.25, 0.57) | ***<0.001*** | -0.32 (-0.48, -0.13) | ***0.011*** |
| VEGF | 106 | 0.05 (-0.14, 0.24) | 0.75 | 0.48 (0.32, 0.62) | ***<0.001*** | -0.19 (-0.37, 0.00) | 0.13 |
| vWF | 106 | 0.12 (-0.07, 0.30) | 0.44 | 0.51 (0.35, 0.64) | ***<0.001*** | -0.23 (-0.40, -0.04) | 0.076 |
| **Plasma Biomarkers** |  |  |  |  |  |  |  |
| A2Macro | 106 | 0.01 (-0.18, 0.20) | 0.92 | 0.16 (-0.03, 0.34) | 0.27 | -0.02 (-0.21, 0.17) | 0.94 |
| AAT | 106 | -0.09 (-0.27, 0.11) | 0.56 | -0.08 (-0.27, 0.11) | 0.61 | 0.21 (0.02, 0.38) | 0.100 |
| ApoAI | 106 | -0.64 (-0.74, -0.51) | ***<0.001*** | -0.01 (-0.20, 0.18) | 0.92 | NA |  |
| B2M | 106 | 0.25 (0.06, 0.42) | 0.052 | 0.05 (-0.14, 0.24) | 0.80 | -0.33 (-0.49, -0.15) | ***0.006*** |
| BDNF | 106 | 0.03 (-0.16, 0.22) | 0.88 | -0.17 (-0.35, 0.03) | 0.25 | 0.06 (-0.13, 0.25) | 0.73 |
| C3 | 106 | 0.33 (0.15, 0.49) | ***0.005*** | 0.01 (-0.18, 0.20) | 0.92 | -0.17 (-0.35, 0.02) | 0.18 |
| CRP | 106 | 0.10 (-0.10, 0.28) | 0.54 | 0.03 (-0.16, 0.22) | 0.86 | -0.21 (-0.39, -0.02) | 0.098 |
| CCL11 | 106 | 0.17 (-0.03, 0.35) | 0.29 | 0.00 (-0.19, 0.19) | 0.97 | -0.15 (-0.33, 0.04) | 0.23 |
| Factor VII | 106 | 0.10 (-0.10, 0.28) | 0.54 | -0.05 (-0.24, 0.15) | 0.80 | -0.01 (-0.21, 0.18) | 0.95 |
| Fibrinogen | 106 | 0.26 (0.07, 0.43) | ***0.045*** | 0.09 (-0.10, 0.28) | 0.56 | -0.16 (-0.34, 0.03) | 0.20 |
| Ferritin | 106 | 0.35 (0.18, 0.51) | ***0.003*** | 0.19 (-0.00, 0.37) | 0.15 | -0.28 (-0.45, -0.10) | ***0.033*** |
| Haptoglobin | 106 | -0.14 (-0.32, 0.05) | 0.37 | 0.09 (-0.10, 0.28) | 0.56 | -0.05 (-0.24, 0.14) | 0.75 |
| ICAM-1 | 106 | 0.01 (-0.18, 0.20) | 0.92 | 0.04 (-0.15, 0.23) | 0.84 | 0.04 (-0.16, 0.22) | 0.85 |
| IL-13 | 106 | -0.37 (-0.52, -0.19) | ***0.002*** | -0.03 (-0.22, 0.16) | 0.86 | 0.26 (0.08, 0.43) | ***0.039*** |
| IL-16 | 106 | 0.30 (0.11, 0.46) | ***0.015*** | 0.09 (-0.10, 0.28) | 0.56 | -0.24 (-0.41, -0.05) | 0.065 |
| IL-18 | 106 | 0.10 (-0.10, 0.28) | 0.54 | 0.12 (-0.07, 0.30) | 0.47 | -0.12 (-0.31, 0.07) | 0.33 |
| IL-3 | 106 | -0.34 (-0.50, -0.16) | ***0.005*** | -0.10 (-0.29, 0.09) | 0.56 | 0.28 (0.09, 0.44) | ***0.033*** |
| IL-6r | 106 | 0.12 (-0.07, 0.30) | 0.44 | 0.06 (-0.13, 0.25) | 0.71 | 0.01 (-0.18, 0.21) | 0.95 |
| IL-8 | 106 | 0.05 (-0.15, 0.23) | 0.77 | 0.14 (-0.06, 0.32) | 0.40 | -0.05 (-0.24, 0.14) | 0.75 |
| CCL2 | 106 | 0.16 (-0.04, 0.34) | 0.32 | -0.13 (-0.31, 0.07) | 0.46 | -0.10 (-0.29, 0.09) | 0.44 |
| MIP-1 alpha | 106 | -0.09 (-0.27, 0.10) | 0.56 | 0.08 (-0.11, 0.27) | 0.60 | -0.10 (-0.28, 0.10) | 0.47 |
| MIP-1 beta | 106 | 0.13 (-0.06, 0.31) | 0.40 | -0.10 (-0.29, 0.09) | 0.56 | -0.04 (-0.23, 0.15) | 0.85 |
| MMP-2 | 106 | -0.15 (-0.33, 0.04) | 0.34 | 0.11 (-0.08, 0.30) | 0.50 | -0.06 (-0.25, 0.13) | 0.73 |
| MMP-9 | 106 | 0.23 (0.04, 0.41) | 0.069 | -0.07 (-0.26, 0.12) | 0.66 | -0.15 (-0.33, 0.05) | 0.23 |
| PAI-1 | 106 | 0.29 (0.11, 0.46) | ***0.018*** | -0.05 (-0.23, 0.15) | 0.80 | -0.15 (-0.33, 0.04) | 0.23 |
| RANTES | 106 | 0.07 (-0.12, 0.26) | 0.65 | -0.12 (-0.30, 0.08) | 0.48 | 0.06 (-0.13, 0.25) | 0.73 |
| SCF | 106 | 0.07 (-0.13, 0.25) | 0.67 | -0.07 (-0.26, 0.12) | 0.66 | -0.05 (-0.24, 0.14) | 0.77 |
| TNF-alpha | 106 | -0.02 (-0.21, 0.17) | 0.92 | -0.03 (-0.22, 0.16) | 0.86 | 0.03 (-0.16, 0.22) | 0.91 |
| TNFR2 | 106 | 0.22 (0.03, 0.39) | 0.097 | 0.09 (-0.10, 0.28) | 0.56 | -0.37 (-0.52, -0.19) | ***0.003*** |
| VCAM-1 | 106 | 0.12 (-0.07, 0.31) | 0.44 | 0.07 (-0.13, 0.25) | 0.69 | -0.15 (-0.33, 0.05) | 0.23 |
| VEGF | 106 | 0.14 (-0.05, 0.32) | 0.37 | 0.03 (-0.16, 0.22) | 0.86 | -0.19 (-0.37, 0.00) | 0.13 |
| vWF | 106 | 0.17 (-0.02, 0.35) | 0.29 | 0.09 (-0.10, 0.28) | 0.56 | -0.20 (-0.37, -0.00) | 0.12 |
| rho: Pearson's correlation; CI: confidence interval; p-values are FDR adjusted within metabolic marker | | | | | | | |
