# Supplementary material for: Metabolic syndrome biomarkers relate to rate of cognitive decline in MCI and dementia stages of Alzheimer’s disease
Source: Alzheimers Res Ther. 2023 Mar 16;15:54. doi: 10.1186/s13195-023-01203-y (PMC10018847; doi:10.1186/s13195-023-01203-y)
Supplement: Supplementary file 3 — Additional file 3: Supplementary Table 2. Associations between TG/HDL, APOA1 and AD and inflammatory biomarkers in Dementia patients [file 13195_2023_1203_MOESM3_ESM.docx]

| **Supplementary Table 2. Associations between TG/HDL, APOA1 and AD and inflammatory biomarkers in Dementia patients** | | | | | | | |
| --- | --- | --- | --- | --- | --- | --- | --- |
|  |  | **Plasma TG/HDL** | | **CSF ApoAI** | | **Plasma ApoAI** | |
| **Factor** | **N** | **rho (95% CI)** | **p-value** | **rho (95% CI)** | **p-value** | **rho (95% CI)** | **p-value** |
| **CSF Markers** |  |  |  |  |  |  |  |
| A-Beta42 | 50 | 0.01 (-0.27, 0.29) | 0.97 | 0.32 (0.05, 0.55) | 0.069 | -0.05 (-0.32, 0.23) | 0.97 |
| P-Tau | 49 | -0.09 (-0.36, 0.20) | 0.75 | -0.06 (-0.34, 0.22) | 0.86 | -0.03 (-0.31, 0.26) | 0.97 |
| Tau | 49 | -0.07 (-0.35, 0.21) | 0.79 | 0.00 (-0.28, 0.28) | 0.99 | -0.04 (-0.32, 0.25) | 0.97 |
| A2Macro | 50 | 0.20 (-0.09, 0.45) | 0.49 | 0.65 (0.45, 0.78) | ***<0.001*** | -0.27 (-0.51, 0.01) | 0.41 |
| AAT | 50 | -0.15 (-0.41, 0.14) | 0.60 | 0.54 (0.30, 0.71) | ***<0.001*** | 0.17 (-0.12, 0.42) | 0.63 |
| ApoAI | 50 | -0.20 (-0.46, 0.08) | 0.49 | NA |  | 0.06 (-0.22, 0.33) | 0.97 |
| B2M | 50 | 0.01 (-0.27, 0.29) | 0.97 | 0.49 (0.25, 0.68) | ***0.001*** | -0.04 (-0.31, 0.24) | 0.97 |
| C3 | 50 | 0.12 (-0.16, 0.39) | 0.69 | 0.50 (0.25, 0.68) | ***0.001*** | -0.32 (-0.55, -0.05) | 0.31 |
| CRP | 50 | 0.11 (-0.17, 0.38) | 0.72 | 0.83 (0.72, 0.90) | ***<0.001*** | -0.22 (-0.47, 0.06) | 0.55 |
| ICAM1 | 50 | -0.00 (-0.28, 0.28) | 0.98 | 0.55 (0.32, 0.72) | ***<0.001*** | -0.02 (-0.29, 0.26) | 0.97 |
| IL16 | 50 | 0.01 (-0.27, 0.29) | 0.97 | 0.46 (0.20, 0.65) | ***0.003*** | -0.02 (-0.30, 0.26) | 0.97 |
| IL25 | 50 | 0.27 (-0.01, 0.51) | 0.27 | 0.20 (-0.08, 0.45) | 0.37 | -0.15 (-0.41, 0.14) | 0.65 |
| IL3 | 50 | 0.10 (-0.18, 0.37) | 0.72 | 0.55 (0.32, 0.72) | ***<0.001*** | -0.21 (-0.46, 0.07) | 0.55 |
| IL6r | 50 | -0.03 (-0.31, 0.25) | 0.95 | 0.24 (-0.04, 0.49) | 0.22 | -0.01 (-0.29, 0.27) | 0.98 |
| IL8 | 50 | -0.03 (-0.31, 0.25) | 0.95 | 0.58 (0.36, 0.74) | ***<0.001*** | 0.01 (-0.27, 0.29) | 0.98 |
| MCP1 | 50 | 0.18 (-0.11, 0.43) | 0.52 | 0.19 (-0.09, 0.45) | 0.37 | -0.12 (-0.38, 0.16) | 0.80 |
| MCP2 | 50 | 0.05 (-0.24, 0.32) | 0.92 | -0.04 (-0.31, 0.24) | 0.88 | 0.33 (0.06, 0.56) | 0.31 |
| MIP1B | 50 | 0.26 (-0.02, 0.51) | 0.27 | 0.19 (-0.09, 0.45) | 0.37 | -0.19 (-0.44, 0.10) | 0.62 |
| MMIF | 50 | 0.16 (-0.12, 0.42) | 0.56 | 0.33 (0.06, 0.56) | 0.064 | -0.15 (-0.41, 0.14) | 0.65 |
| MMP2 | 50 | 0.20 (-0.09, 0.45) | 0.49 | 0.38 (0.12, 0.60) | ***0.021*** | -0.20 (-0.45, 0.08) | 0.55 |
| MMP3 | 50 | 0.01 (-0.27, 0.29) | 0.97 | 0.02 (-0.26, 0.30) | 0.93 | 0.04 (-0.24, 0.32) | 0.97 |
| PAI1 | 50 | -0.02 (-0.29, 0.26) | 0.97 | 0.59 (0.37, 0.74) | ***<0.001*** | 0.08 (-0.21, 0.35) | 0.97 |
| SCF | 50 | 0.18 (-0.10, 0.44) | 0.51 | 0.24 (-0.04, 0.49) | 0.22 | -0.24 (-0.48, 0.04) | 0.51 |
| TIMP1 | 50 | -0.10 (-0.36, 0.19) | 0.72 | 0.65 (0.45, 0.79) | ***<0.001*** | 0.02 (-0.26, 0.29) | 0.97 |
| VCAM1 | 50 | -0.09 (-0.36, 0.19) | 0.72 | 0.62 (0.41, 0.77) | ***<0.001*** | -0.02 (-0.30, 0.26) | 0.97 |
| VEGF | 50 | 0.09 (-0.20, 0.36) | 0.75 | 0.41 (0.15, 0.62) | ***0.011*** | -0.05 (-0.32, 0.23) | 0.97 |
| vWF | 50 | 0.15 (-0.14, 0.41) | 0.60 | 0.45 (0.20, 0.65) | ***0.003*** | -0.16 (-0.42, 0.12) | 0.63 |
| **Plasma Biomarkers** |  |  |  |  |  |  |  |
| A2Macro | 50 | -0.08 (-0.35, 0.20) | 0.75 | 0.02 (-0.26, 0.29) | 0.94 | 0.25 (-0.04, 0.49) | 0.51 |
| AAT | 50 | -0.11 (-0.37, 0.18) | 0.72 | 0.32 (0.05, 0.55) | 0.069 | 0.16 (-0.12, 0.42) | 0.63 |
| ApoAI | 50 | -0.64 (-0.78, -0.44) | ***<0.001*** | 0.06 (-0.22, 0.33) | 0.86 | NA |  |
| B2M | 50 | 0.39 (0.12, 0.60) | 0.048 | -0.00 (-0.28, 0.27) | 0.99 | -0.10 (-0.37, 0.18) | 0.84 |
| BDNF | 50 | 0.11 (-0.17, 0.38) | 0.72 | 0.09 (-0.19, 0.36) | 0.76 | -0.24 (-0.49, 0.04) | 0.51 |
| C3 | 50 | 0.39 (0.13, 0.60) | ***0.048*** | 0.04 (-0.24, 0.32) | 0.88 | -0.30 (-0.53, -0.02) | 0.39 |
| CRP | 50 | 0.19 (-0.09, 0.45) | 0.49 | 0.13 (-0.16, 0.39) | 0.60 | -0.42 (-0.62, -0.16) | 0.068 |
| CCL11 | 50 | 0.39 (0.12, 0.60) | ***0.048*** | 0.05 (-0.23, 0.32) | 0.88 | -0.00 (-0.28, 0.28) | 0.99 |
| Factor VII | 50 | 0.34 (0.07, 0.56) | 0.11 | -0.25 (-0.50, 0.03) | 0.22 | 0.11 (-0.17, 0.38) | 0.80 |
| Fibrinogen | 50 | 0.06 (-0.23, 0.33) | 0.88 | 0.08 (-0.21, 0.35) | 0.81 | 0.00 (-0.28, 0.28) | 0.99 |
| Ferritin | 50 | 0.12 (-0.16, 0.39) | 0.69 | 0.15 (-0.14, 0.41) | 0.52 | -0.17 (-0.43, 0.11) | 0.63 |
| Haptoglobin | 50 | 0.16 (-0.12, 0.42) | 0.56 | -0.19 (-0.45, 0.09) | 0.37 | -0.11 (-0.37, 0.18) | 0.83 |
| ICAM-1 | 50 | 0.30 (0.02, 0.53) | 0.21 | 0.04 (-0.24, 0.31) | 0.88 | -0.15 (-0.41, 0.14) | 0.65 |
| IL-13 | 50 | -0.13 (-0.39, 0.15) | 0.68 | -0.15 (-0.41, 0.14) | 0.52 | 0.11 (-0.17, 0.38) | 0.80 |
| IL-16 | 50 | 0.10 (-0.19, 0.36) | 0.72 | 0.08 (-0.20, 0.35) | 0.79 | 0.05 (-0.23, 0.32) | 0.97 |
| IL-18 | 50 | 0.34 (0.07, 0.56) | 0.11 | 0.12 (-0.16, 0.39) | 0.60 | -0.18 (-0.44, 0.10) | 0.63 |
| IL-3 | 50 | -0.69 (-0.81, -0.51) | ***<0.001*** | 0.22 (-0.07, 0.47) | 0.30 | 0.57 (0.35, 0.73) | ***<0.001*** |
| IL-6r | 50 | 0.04 (-0.24, 0.32) | 0.92 | -0.09 (-0.36, 0.20) | 0.79 | 0.03 (-0.25, 0.30) | 0.97 |
| IL-8 | 50 | 0.19 (-0.10, 0.44) | 0.49 | 0.30 (0.02, 0.53) | 0.098 | -0.02 (-0.29, 0.26) | 0.97 |
| CCL2 | 50 | 0.21 (-0.07, 0.46) | 0.49 | 0.24 (-0.04, 0.49) | 0.22 | -0.18 (-0.43, 0.11) | 0.63 |
| MIP-1 alpha | 50 | 0.15 (-0.13, 0.41) | 0.60 | 0.25 (-0.03, 0.49) | 0.22 | -0.05 (-0.33, 0.23) | 0.97 |
| MIP-1 beta | 50 | 0.19 (-0.10, 0.44) | 0.49 | 0.18 (-0.10, 0.44) | 0.39 | -0.21 (-0.46, 0.07) | 0.55 |
| MMP-2 | 50 | -0.20 (-0.45, 0.08) | 0.49 | 0.07 (-0.21, 0.35) | 0.81 | 0.27 (-0.01, 0.51) | 0.41 |
| MMP-9 | 50 | 0.10 (-0.18, 0.37) | 0.72 | 0.14 (-0.14, 0.40) | 0.54 | -0.05 (-0.32, 0.23) | 0.97 |
| PAI-1 | 50 | 0.27 (-0.01, 0.51) | 0.27 | 0.02 (-0.26, 0.30) | 0.93 | -0.28 (-0.51, 0.00) | 0.41 |
| RANTES | 50 | -0.02 (-0.30, 0.26) | 0.97 | 0.15 (-0.14, 0.41) | 0.52 | -0.11 (-0.38, 0.17) | 0.80 |
| SCF | 50 | 0.34 (0.07, 0.57) | 0.11 | -0.02 (-0.30, 0.26) | 0.93 | -0.20 (-0.46, 0.08) | 0.55 |
| TNF-alpha | 50 | 0.17 (-0.12, 0.43) | 0.55 | 0.15 (-0.14, 0.41) | 0.52 | -0.04 (-0.31, 0.24) | 0.97 |
| TNFR2 | 50 | 0.28 (-0.00, 0.52) | 0.27 | 0.14 (-0.14, 0.41) | 0.52 | -0.05 (-0.33, 0.23) | 0.97 |
| VCAM-1 | 50 | 0.01 (-0.27, 0.29) | 0.97 | 0.07 (-0.21, 0.34) | 0.83 | 0.07 (-0.21, 0.34) | 0.97 |
| VEGF | 50 | 0.53 (0.30, 0.71) | ***<0.001*** | 0.05 (-0.23, 0.32) | 0.88 | -0.21 (-0.46, 0.08) | 0.55 |
| vWF | 50 | 0.20 (-0.08, 0.45) | 0.49 | 0.05 (-0.23, 0.33) | 0.88 | -0.16 (-0.42, 0.13) | 0.64 |
| rho: Pearson's correlation; CI: confidence interval; p-values are FDR adjusted within metabolic marker | | | | | | | |
